# Surgical site infection and pathogens in Ethiopia: a systematic review and meta-analysis

**DOI:** 10.1186/s13037-020-00232-y

**Published:** 2020-02-21

**Authors:** Yeneabat Birhanu, Aklilu Endalamaw

**Affiliations:** 1 Department of Surgical Nursing, School of Nursing, College of Medicine and Health Sciences, University of Gondar, Gondar, Ethiopia; 2 Department of Pediatrics and Child Health Nursing, School of Health Sciences, College of Medicine and Health Sciences, Bahir Dar University, Bahir Dar, Ethiopia

**Keywords:** Bacteria, Pathogen, Surgical site infection, Ethiopia

## Abstract

**Background:**

Surgical site infection is a common complication in patients who underwent surgery. The prevalence is higher in low-income countries. In Ethiopia, prevalence and pathogens of surgical site infection (SSI) reported are variable. This systematic review and meta-analysis aimed to find the pooled prevalence of SSI. Besides, it aimed to find pathogens of surgical site infection in Ethiopia.

**Methods:**

The databases for the search were PubMed, Web of Science, and Google Scholar by the date 21/08/2018. To assess publication bias Egger’s test regression analysis was applied. Subgroup analysis was conducted based on the study population and region.

**Results:**

This meta-analysis included a total of 15 studies with 8418 study subjects. The pooled prevalence of surgical site infection was 25.22% (95% CI: 17.30 to 33.14%). *Staphylococcus aureus* (30.06%) was the most common pathogen identified. Followed by *Escherichia coli* (19.73%), *Klebsiella species* (17.27%), and *Coagulase-Negative staphylococci* (12.43%) were the commonly isolated pathogens.

**Conclusions:**

The national prevalence of surgical site infection was high. The most common identified pathogen was *Staphylococcus aureus*. Followed by *Escherichia coli*, *Klebsiella species,* and Coagulase-Negative staphylococci. Strict adherence to surgical site infection prevention techniques needs to get more attention.

## Background

Surgical site infection (SSI) is one of the global health problems [1].. It contributes to occur antibiotic resistance that further leads to life-threatening morbidity [2]. It also increases hospital stay and costs of healthcare services [3, 4]. Besides, it surges the economic burden and impaired quality of life of the patients [5]. Moreover, according to the CDC report, SSI causes 3% of death by the end of 2015 [1].

Effective infection prevention activities have implemented in national and international settings. These are because of to prevent and control devastating health problems. Notable, improving surgical techniques, operating rooms, and providing antimicrobial prophylaxis [6]. The others include decontamination, preoperative bathing, decolonization with mupirocin ointment, enhancing nutrition [7]. Yet, surgical site infection remains one of the common causes of healthcare-associated infection.

A first global report claimed that SSI is one of the most major problems. This has shown that it occurred in every type of surgical procedure [8]. According to the 2016 WHO report, after the surgery infected patients were 11% [7]. Its burden reported both in developed and developing countries. In mainland China, SSI reported as 4.5% [9],14.8% in sub-Sahara Africa [10], 16.4% in Uganda [11], and 13.0–22.05% in Nigeria [12, 13]. Another systematic review study reported the burden of bacterial pathogens. The most common isolated organism for SSI was *Staphylococcus aureus* (*S. aureus* (30.4%) [4]. *Coagulase-Negative staphylococci* (*CONS*) (11.7%), *E. coli* (9.4%), *E. faecalis* (5.9%), *Pseudomonas aeruginosa* (5.5%),*Enterobacter species* (4.0%), and *Klebsiella species* (4.0%) were also isolated [7].

In Ethiopia, different studies had conducted to find the prevalence of SSI and pathogens. The prevalence of SSI found in the range between 6.4 to 75.5% [14, 15] in the Ethiopian setting. Hence, discrepancies between studies make difficult to represent the national prevalence. Having national representative data is real to underpin effective prevention and control strategies. Thus, a need to have a pooled estimation of SSI recognizes at the country level. This systematic review and meta-analysis aimed to find the pooled prevalence of SSI. Besides, it aimed to find pathogens of SSI in the Ethiopian setting. The review question was what are the prevalence and pathogens for SSI in Ethiopia?

## Methods

### Reporting

The Preferred Reporting Items for Systematic Reviews and Meta-analyses (PRISMA) guideline [16] was used to report this meta-analysis (Additional file 1 research checklist).

### Literature search

The databases for the search were Medline (PubMed), Web of Science, and Google Scholar databases. The terms for the search were pre-defined for a comprehensive search strategy. These included all fields within records and Medical Subject Headings (MeSH terms). In the Boolean operator, within each axis, we combined keywords with the “OR” operator. Then we linked the search strategies for the two axes with the “AND” operator. The search terms used for the search were “Surgical site infection” OR “hospital-acquired infection” OR “nosocomial infection” OR “wound site infection” OR “surgical wound site infection” AND “prevalence” OR “distribution” OR “incidence” OR “burden” OR “epidemiology” AND “pathogens” OR “bacteria” AND “Ethiopia”. The specific searching detail in PubMed with MeSH terms was (“prevalence of surgical site infection” [MeSH Terms] OR “surgical site infection” [MeSH Terms] OR “hospital-acquired infection” [MeSH Terms] OR “nosocomial infection” [MeSH Terms] OR “wound site infection” [MeSH Terms] OR “surgical wound site infection” [MeSH Terms] AND “prevalence” [All Fields]) OR “distribution” [MeSH Terms] OR “incidence” [MeSH Terms] OR “burden” [MeSH Terms] OR “epidemiology” [MeSH Terms] AND pathogens of surgical site infection [All Fields]) AND (“Ethiopia” [MeSH Terms] by the date 21/08/2018. The publication year of the studies was not limited during the search.

### Study selection

All retrieved studies were exported to Endnote version 7 (Thomson Reuters, London) reference manager. It is the study selection method that we used to remove duplicated studies.

The retrieved articles were screened according to pre-defined inclusion and exclusion criteria. Discussion and/or involvement of the third reviewer resolved any disagreements.

### Eligibility criteria

#### Inclusion criteria

Included studies were articles that reported the prevalence of SSI and/or bacterial pathogens. It also included studies published in English and studies conducted only in Ethiopia.

#### Exclusion criteria

Excluded criteria were articles without full-text available and qualitative studies. Other excluded criteria were any reviews, commentaries, consultants’ corners, letters, and conference abstracts.

### Quality assessment

We used Joanna Brigg’s Institute (JBI) quality appraisal criteria [17].. It is the assessment tool used to check the quality of each article. The tool consists of nine major items. The first item is appropriate to the sample frame. The second is the appropriate sampling technique. The third is the adequacy of the sample size. The fourth is a description of the study subjects and settings. The fifth is enough coverage of data analysis. The sixth is the validity of the method for identification of the condition. The seventh item is a standard and reliable measurement for all participants. The eighth is the appropriateness of statistical analysis. And the last item is adequacy and management of response rate. Studies considered low-risk when it would fit 5 or above quality assessment checklists.

### Data extraction

A standardized form was used to extract data by two authors. The following information from each article was extracted. Such as first author, and publication year, the study design, and study population. The location of the study and the type of bacteria were also extracted.

### Outcome measurement

This systematic review and meta-analysis have two major outcomes. The first outcome is to determine the prevalence of SSI in Ethiopia. It calculated as dividing the number of patients who develop SSI to the total number of patients multiply by 100. A total number of patients refer to patients underwent surgery in the study period. The second outcome of the study was to identify the pathogens of surgical site infection. SSI is an infection where a person presents with signs and symptoms of the infection [18, 19].

### Data analysis

The required data were collected using a Microsoft Excel 2010 workbook form. Used to collect the required data. Then, the STATA Version11 software was used to analyze the data. The original articles presented using tables and forest plots. A weighted inverse variance random-effects model [20] used to estimate the pooled prevalence. I^2^ statistics used to assess the percentage of total variation across studies [21]. I^2^ ≤ 25% suggested more homogeneity,25% < I^2^ ≤ 75% suggested moderate heterogeneity, and I^2^ > 75% suggested high heterogeneity [21]. Egger’s regression test was also used to assess publication bias [22]. Furthermore, the sub-group analysis carried out based on the region of studies. This reduces the random discrepancies between the point estimates of the primary study.

## Results

### Literature search result

**Fig. 1:**
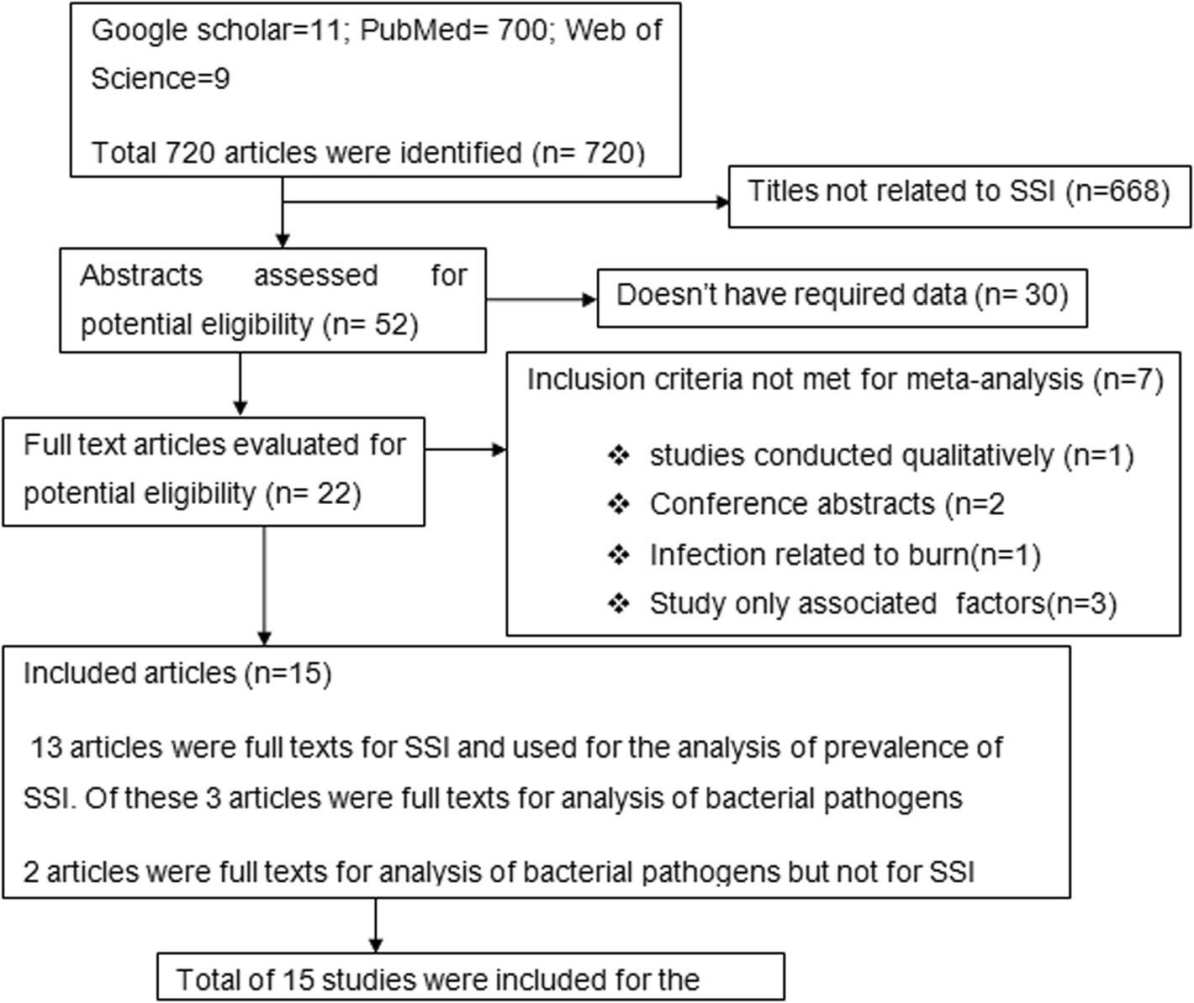
Flow chart of the literature search of articles included in a meta-analysis of SSI

A comprehensive literature search of the database yielded a total of 720 publications. Among these, 705 disregarded due to qualitative study, abstracts, conference abstracts, and titles. Of 15 eligible studies, identified for SSI were 13 studies [14, 15, 23–33] with 7786 study participants. Studies identified for pathogens were Five studies [15, 25, 28, 34, 35] with 629 subjects. These five studies conducted on patients who already developed surgical site infections. Finally, this meta-analysis includes a total of 15 studies with 8418 subjects [14, 15, 23–35] (Fig. 1).

### Characteristics of included studies

The range of publication year for included studies was from 2005 to 2018. Four regions and Addis Ababa, the capital city of Ethiopia was the settings studies found. Five in Southern Nation, Nationalities, and People Region (SNNPR) [29, 30, 33–35], three in Addis Ababa [15, 23, 28], three in Amhara [24, 26, 27], two in Oromia [31, 32], and two in Tigray region [14, 25]. All included studies were done by using the cross-sectional study design (Table 1).

**Table 1:** Characteristics of included studies in the meta-analysis of SSI

Author/Year	Study year	Region	Study design	Sample size	Prevalence
Gelaw KA et al./2017 [14]	July 2013–June 2016	Tigray	Cross-sectional	384	6.4
Asres G et al./2017 [15]	March–August 2015	Addis Ababa	Cross-sectional	197	75.5
Taye M/2005 [23]	January 1999–December 1999	Addis Ababa	Cross-sectional	1754	14.8
Melaku S et al./2012 [24]	April–August 2009	Amhara	Cross-sectional	961	17.1
Mengasha RE et al./2014 [25]	January–June 2012	Tigray	Cross-sectional	128	75
Gelaw A et al./2014 [26]	November 2010–February 2011	Amhara	Cross-sectional	510	8.2
Yalew WW et al./2016 [27]	March–April and July 2015	Amhara	Cross-sectional	908	51.1
Dessie W et.al/2016 [28]	October 2013–March 2014	Addis Ababa	Cross-sectional	1088	9.8
Wodajo S et al./2017 [29]	June 2012–May 2013	SNNP	Cross-sectional	592	11
Laloto TL et.al/2017 [30]	March 2–May 2, 2015	SNNP	Cross-sectional	105	19.1
Mamo T et al./2017 [31]	May–September 2015	Oromia	Cross-sectional	384	9.4
Ali S et al./2018 [32]	May–September 2016	Oromia	Cross-sectional	450	22
Dacho AM/2018 [33]	March 10–30, 2017	SNNP	Cross-sectional	325	12.9

Five of the studies have reported isolated bacterial pathogens from SSI [15, 25, 28, 34, 35] (Additional file 1)**.**

We did an assessment of studies with JBI quality appraisal checklists. Based on this, none of the included studies was poor quality status.

### Meta-analysis

The absence of publication bias was assessed with Egger’s regression test (*p* = 0.068), which showed that no publication bias.

**Fig. 2:**
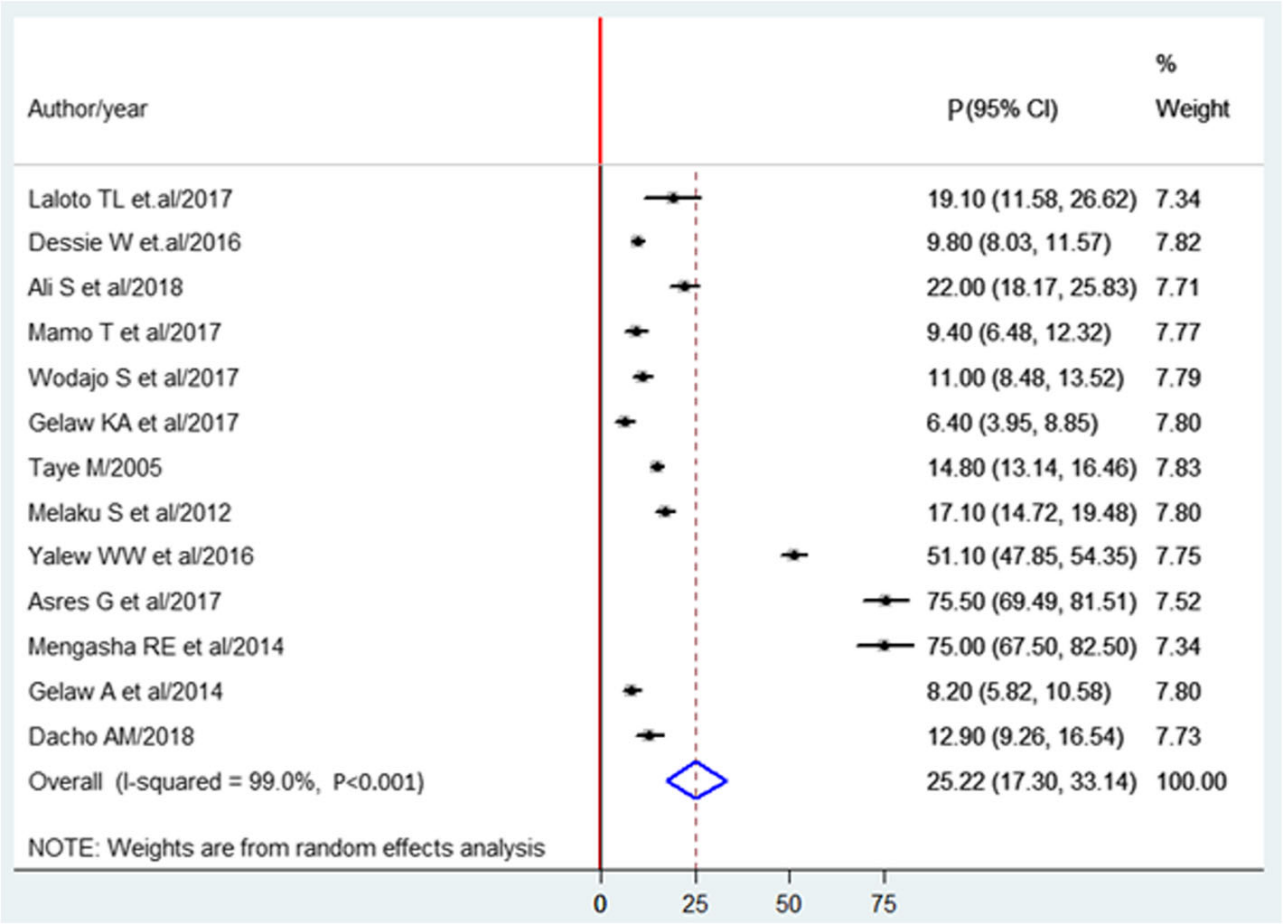
Forest plot of prevalence with corresponding 95% CIs of the thirteen studies on SSI. The midpoint and the length of each segment indicated prevalence and a 95% CI. The diamond shape showed the combined prevalence of all studies

**Fig. 3:**
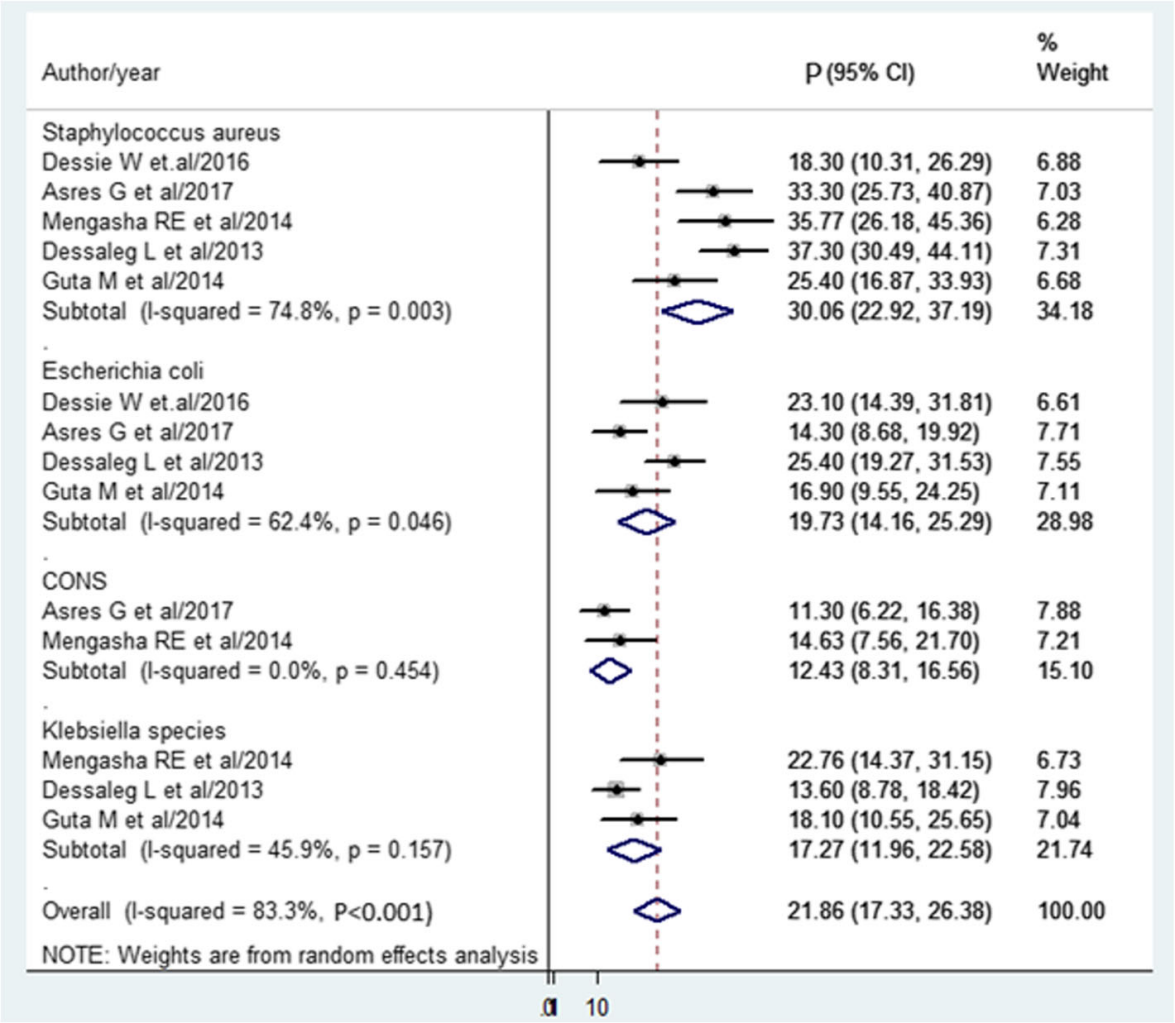
Forest plot of prevalence with corresponding 95% CIs of pathogens. The midpoint and the length of each segment indicated prevalence and a 95% CI. The diamond shape showed the combined prevalence of all studies

The pooled prevalence of SSI estimated from 13 studies [14, 15, 23–33] was 25.22%(95% CI, 17.30 to 33.14%) (Fig. 2). The pooled prevalence of pathogens from five studies [15, 25, 28, 34, 35] showed that *S. aureus* (30.6%) was most prevalent. Followed by *E.coli* (19.73%), Klebsiella spp. (17.27%), *and CONS* (12.43%) (Fig. 3).

### Subgroup analysis

**Fig. 4:**
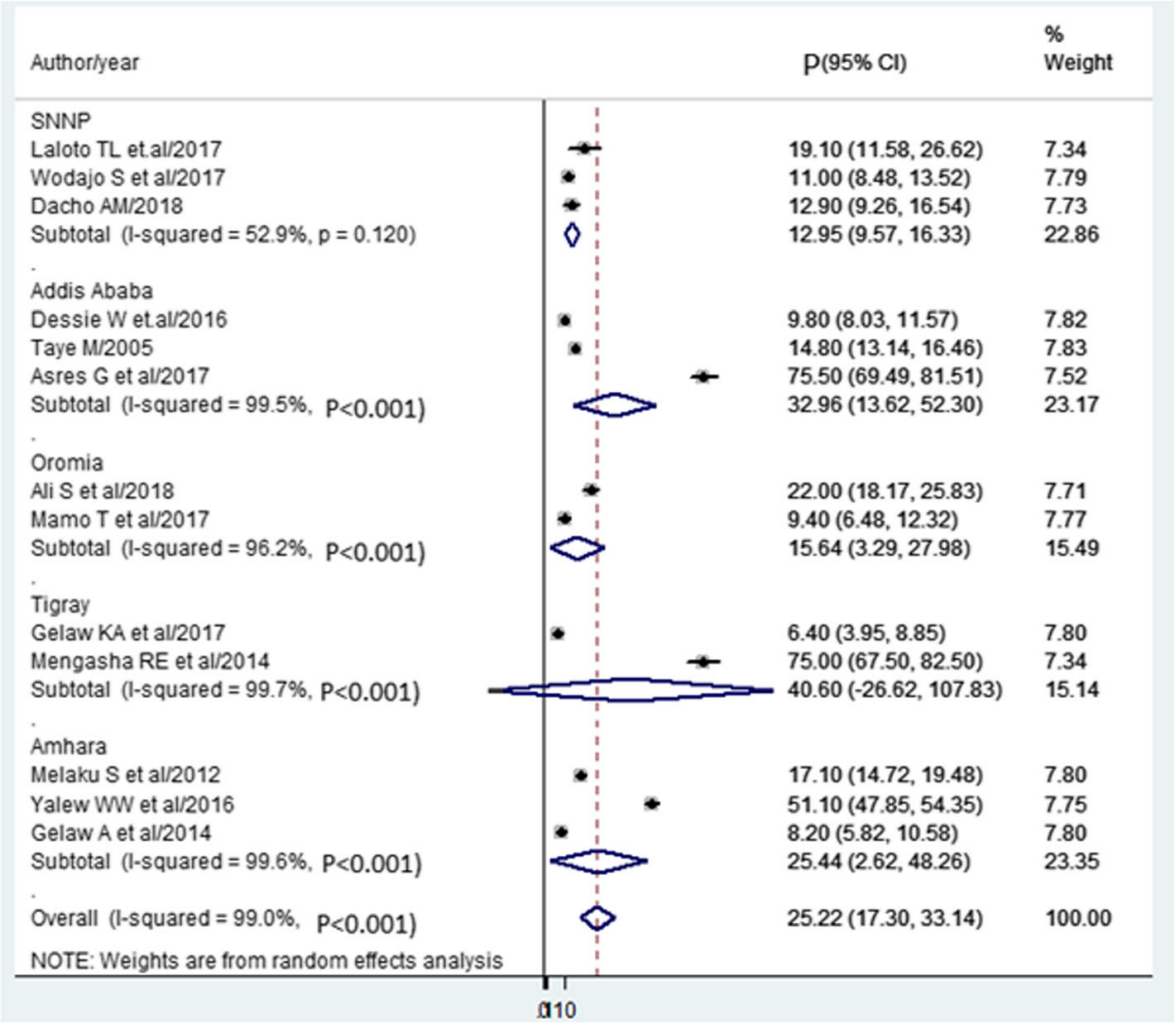
Forest plot of prevalence with corresponding 95% CIs on the region. The midpoint and the length of each segment indicated prevalence and a 95% CI. The diamond shape showed the combined prevalence of all studies

Based on the subgroup analysis, the Tigray region ranked first (40.60%). Followed by Addis Ababa (32.96%), Amhara (25.44%), and Oromia region (15.64%) were the regions ranked. The report of the lowest prevalence of SSI was from the SNNPR (12.95%) (Fig. 4).

**Fig. 5:**
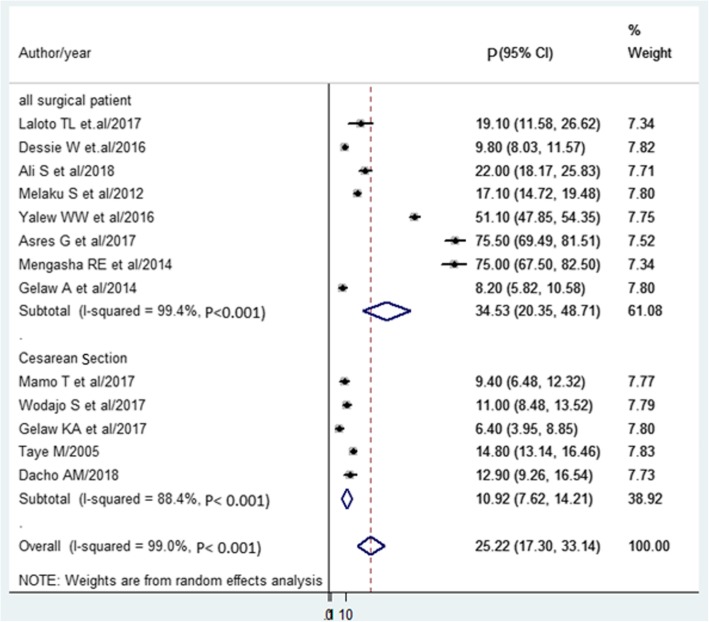
Forest plot of prevalence with corresponding 95% CIs on the type of surgery. The midpoint and the length of each segment indicated prevalence and a 95% CI. The diamond shape showed the combined prevalence of all studies

The subgroup analysis was also done based on the study population. Eight studies done on all surgical patients showed that the prevalence of SSI was 34.53%. The pooled prevalence of mothers with Cesarean Section (CS) found to be 10.92% (Fig. 5).

## Discussion

Surgical site infection continues a global burden of infectious diseases. Particularly, it is common infectious diseases in resource-limited countries including Ethiopia [14, 15].

According to this meta-analysis, the estimation of SSI found was 25.22% (17.30, 33.14) in Ethiopia. This is comparable with the study conducted in Tanzania [36] and Nigeria [13]. Factors of surgical site infection are almost similar in developing countries [37]. Besides, infection prevention methods or surgical settings might be similar in developing countries.

This study is higher than a study conducted in Burundi and the Democratic Republic of Congo [38]. This discrepancy might be due to the difference in the study population. In the current study, the prevalence of SSI estimated from all surgical patients. In later, the SSI estimated from only the CS cases.

The current finding is higher than the study from Mainland China [9] and Southeast Asia [39]. This difference might be due to the lack of infection control guidelines. Evidence shows that prophylaxis often administered too early or too late during surgery. This is the condition that, prophylaxis to be ineffective in reducing patient harm [40, 41]. Moreover, negligence [42], improper sterilization [43], poor hand hygiene [44] might increases SSI. But not preoperative bathing, enhanced nutritional support, perioperative discontinuation of immunosuppressive agents [45].

Based on the subgroup analysis, the regional prevalence of SSI was also determined. The highest prevalence of SSI noted in the Tigray region of Ethiopia (40.6%). This is almost three times higher than a result of Southern Ethiopia (12.95%). This might be the study conducted in the Tigray region was teaching hospitals. But, the studies done in Southern Ethiopia were district hospitals. This is the site designed to give only healthcare service rather than teaching.

Based on bacterial pathogen estimation, the most common identified pathogen was *S. aureus.* The same report from India [46], Nigeria [12], and Uganda [47] showed that *S.aureus* found the most common causes of SSI. This might be due to *S.aureus* is part of human skin normal flora [48]. So, during the surgical procedure, it could enter into the internal surface of the body. This finding helps healthcare policy and/or decision-makers to consider SSI prevention principles.

Due to the lack of studies in some locations of Ethiopia, the result may not represent a national figure. Although *I*^*2*^ is not an absolute measure of heterogeneity, high heterogeneity was observed.

## Conclusions

In this finding, the prevalence of SSI was higher compared to the standard CDC guidelines for SSI [49]. Tigray region ranked first followed by Addis Ababa, Amhara, and Oromia region. While the lowest prevalence observed in the Southern region. The most common identified pathogen of SSI *was Staphylococcus aureus*. Followed by identified pathogens are *Escherichia coli*, *Klebsiella spp,* and *CONS*. Thus, efforts should make to ensure the prevention of surgical site infection. It means healthcare facilities give more emphasis on infection control measures. Furthermore, effective pre- and post-operative antibiotics should give to patients undergoing surgery. Finally, active SSI surveillance and infection prevention strategies must be established at the national level.

## Supplementary information

**Additional file 1.** Research checklist. Characteristics of included studies in the meta-analysis of bacterial pathogens.

## Data Availability

No need of more data. All information stated in the manuscript and, its supplementary information files.

## Abbreviations

CI: Confidence Interval
CONS: Coagulase-Negative staphylococci
SNNPR: Southern Nations and Nationalities of People Region
SPP: Species
SSI: Surgical Site Infection
