# Supplementary material for: Surgical site infection and pathogens in Ethiopia: a systematic review and meta-analysis
Source: Patient Saf Surg. 2020 Feb 21;14:7. doi: 10.1186/s13037-020-00232-y (PMC7035652; doi:10.1186/s13037-020-00232-y)
Supplement: Supplementary file 1 — Additional file 1. Research checklist. Characteristics of included studies in the meta-analysis of bacterial pathogens. [file 13037_2020_232_MOESM1_ESM.zip › Additional file 1.docx]

| Author/year | Study year | Region | Study design | Sample size | Pathogen type | prevalence% | Surgery type |
| --- | --- | --- | --- | --- | --- | --- | --- |
| Dessie W et.al/2016 [[28](#_ENREF_28)] | October 2013 - March 2014 | Addis Ababa | Cross-sectional | 90 | S. aureus | 18.3 | All surgical patient |
|  |  |  |  |  | E. coli | 23.1 |  |
| Asres G et al/2017[[15](#_ENREF_15)] | March - August 2015 | Addis Ababa | Cross-sectional | 149 | S. aureus | 33.3 | All surgical patient |
|  |  |  |  |  | E. coli | 14.3 |  |
|  |  |  |  |  | CONS | 11.3 |  |
| Mengasha RE et al/2014[[25](#_ENREF_25)] | January -  June 2012 | Tigray | Cross-sectional | 96 | S. aureus | 35.77 | All surgical patient |
|  |  |  |  |  | Klebsiella species | 22.76 |  |
|  |  |  |  |  | CONS | 14.63 |  |
| Dessalegn L et al/2013[[34](#_ENREF_34)] | November 2010 - March 2011 | SNNP | Cross-sectional | 194 | S. aureus | 37.3 | All surgical patient |
|  |  |  |  |  | E. coli | 25.4 |  |
|  |  |  |  |  | Klebsiella species | 13.6 |  |
| Guta M et al/2014[[35](#_ENREF_35)] | November 2010- June 2011 | SNNP | Cross-sectional | 100 | S. aureus | 25.4 | All surgical patient |
|  |  |  |  |  | Klebsiella spp | 18.1 |  |
|  |  |  |  |  | E. coli | 16.9 |  |
